# Carbon catabolite regulation of secondary metabolite formation, an old but not well‐established regulatory system

**DOI:** 10.1111/1751-7915.13791

**Published:** 2021-03-06

**Authors:** Beatriz Ruiz‐Villafán, Rodrigo Cruz‐Bautista, Monserrat Manzo‐Ruiz, Ajit Kumar Passari, Karen Villarreal‐Gómez, Romina Rodríguez‐Sanoja, Sergio Sánchez

**Affiliations:** ^1^ Instituto de Investigaciones Biomédicas Universidad Nacional Autónoma de México Ciudad Universitaria CdMx México City 04510 México

## Abstract

Secondary microbial metabolites have various functions for the producer microorganisms, which allow them to interact and survive in adverse environments. In addition to these functions, other biological activities may have clinical relevance, as diverse as antimicrobial, anticancer and hypocholesterolaemic effects. These metabolites are usually formed during the idiophase of growth and have a wide diversity in their chemical structures. Their synthesis is under the impact of the type and concentration of the culture media nutrients. Some of the molecular mechanisms that affect the synthesis of secondary metabolites in bacteria (Gram‐positive and negative) and fungi are partially known. Moreover, all microorganisms have their peculiarities in the control mechanisms of carbon sources, even those belonging to the same genus. This regulatory knowledge is necessary to establish culture conditions and manipulation methods for genetic improvement and product fermentation. As the carbon source is one of the essential nutritional factors for antibiotic production, its study has been imperative both at the industrial and research levels. This review aims to draw the utmost recent advances performed to clarify the molecular mechanisms of the negative effect exerted by the carbon source on the secondary metabolite formation, emphasizing those found in *Streptomyces*, one of the genera most profitable antibiotic producers.

## Graphical abstract

Carbon catabolite regulation of secondary metabolite formation. Carbon source regulation of secondary metabolite production. Carbon regulation on secondary metabolites in Streptomyces.

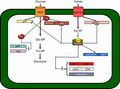

## Introduction

Carbon catabolite repression (CCR) is a general mechanism that governs the sequential use of carbon sources in microorganisms and has a meaningful impact on many other cellular functions. The assembly of regulatory networks coordinates the differential expression of genes under both limiting and non‐limiting carbon source conditions. These mechanisms promote the use of nutrients that support high growth rates and consequently limit the expression of genes that are not essential **f**or growth, preventing high metabolic cost.

The presence of two or more sources of carbon in the growing environment has a relevant impact on growth. The classical example is the study by Monod (1949) on the diauxic growth of *Escherichia coli* observed by the sequential use of glucose and sorbitol. Although many microorganisms use glucose as their preferred carbon source for growth, choosing the preferred source of carbon is a matter of niche. Therefore, bacteria like *Streptococcus thermophilus* prefer lactose over glucose (van Den ogaard *et al*., 2000). In *Pseudomonas aeruginosa,* there is a hierarchy of substratum preferences. It begins with the use of amino acids such as aspartate, followed by citrate, succinate, lactate, acetate, and ultimately glucose, in a strategy known as ‘reverse diauxic’ (Brückner and Titgemeyer, 2002). This distribution of substrate use precludes competition within the microbial community (McGill *et al*., 2021). Yeast could maintain a high fitness under stable glucose conditions by suppressing the costly expression of genes necessary for development in alternative carbon sources. However, the consequence is a slow transcriptional change when glucose depletion occurs, resulting in reduced fitness when adapting to a non‐preferential carbon source. In contrast, less stringent CCR has a fitness cost under stable conditions, but allows for a robust and more uniform adaptation, leading to better fitness in a changing environment (New *et al*., 2014).

Some recent reports on CCR have focused on understanding the use of hardly assimilable substrates such as lignocellulose (Shiwa *et al*., 2020; Zheng *et al*., 2020; Wu *et al*., 2021). However, the mechanism of CCR has effects beyond the use of carbon sources in phenomena such as virulence or the production of secondary metabolites. Secondary metabolites are low molecular mass products like isoprenes, polyketides, aromatic compounds, oligosaccharides and peptides. Their synthesis typically takes place during the microbial idiophase. These metabolites are essential in our modern society because of their broad utilization as antibiotics, anticancer, cholesterol‐lowering drugs, immunosuppressants, antimycotics, anthelmintics, antivirals and others (Sánchez *et al*., 2019). The presence of various carbon sources precludes the synthesis of secondary metabolites (Romero‐Rodríguez et al., 2018). This negative effect is present in fungi and bacteria, although major differences are present in their regulation according to the microorganism under consideration. This review will analyse the main mechanisms of CCR, emphasizing their importance in producing secondary metabolites. Although a great deal of information is available about enteric bacteria, in particular *E. coli*, and Gram‐positive with low GC content, we will mainly focus on our discussion of Gram‐positive bacteria with high GC content.

### Carbon source regulation of secondary metabolite production in Gram‐negative bacteria

For the last 60 years, the classical model used to perform CCR studies has been that of *E. coli*.

This model holds its CCR mechanism on the enzyme IIAglc (EIIAglc) from the phosphoenolpyruvate‐dependent phosphotransferase system (PTS) (Ammar *et al*., 2018). As long as an increase in glucose concentration occurs, the phosphorylated EIIAglc decreases, preventing other sugars uptake, by an effect known as inducer exclusion. On the contrary, in the absence of glucose, EIIAglc remains phosphorylated and activates adenylate cyclase (Cya). This condition allows the transport of other carbon sources and globally modulates the transcription of around 200 genes in these enterobacteria (Pannuri *et al*., 2016; Park *et al*., 2020).

*Serratia marcescens* is a Gram‐negative and opportunistic pathogen. It produces the hydrophobic pigment prodigiosin, a molecule with essential functions in this bacterium. Besides its role of enabling this microorganism to compete against other bacteria, prodigiosin regulates proton gradient or pH homeostasis (Fender et al., 2012). Prodigiosin also presents bioactivities clinically relevant as immunosuppressant or antitumoral. However, its production can be repressed by glucose due to medium acidification and pH decrease. Mutants lacking the quinoprotein glucose dehydrogenase (GdhS) gene (*gcd*) prevent media acidification of fermentation culture. The GdhS enzyme catalyses the first step of glucose utilization in *S. marcescens*, generating D‐glucono‐1,5‐lactone, which produces D‐gluconic acid by non‐enzymatic hydrolysis (Fender *et al*., 2012). Null‐mutants of the *gcd* gene exhibit high transcription levels of the prodigiosin biosynthetic genes. Thus, this mutation favours antibiotic production and prevents the inhibitory effect caused by medium acidification.

One additional example of CCR results from the transcriptomic study performed in the marine microorganisms *Vibrio coralliilyticus* S2052 and *Photobacterium galatheae* S2753, producers of andrimid and holomycin respectively. These microorganisms are inhibitors of the pathogen *Vibrio anguillarum*. An upregulation in andrimid and holomycin synthesis is observed by growing both strains in the presence of glucose and chitin (Giubergia et al., 2017). The positive effect of chitin on andrimid biosynthesis could be due to an upregulation of the transcriptional regulator TW71_08080 (of the LysR family), which lies downstream to the biosynthetic gene cluster (BGC). Similarly, upstream the BGC for holomycin lies the transcriptional regulator EA58_20500 of the AsR family, which is also downregulated by chitin, pointing to a possible repressor role. The positive chitin effect on secondary metabolism is related to both marine strain’s ecological niche, where chitin is a common carbon source (Giubergia et al., 2017).

Although many studies on CCR have used glucose as a preferential carbon source for growth and secondary metabolites production, there are many other microorganisms in which glucose is not the best source. Examples of this are the genera *Acinetobacter* and *Pseudomonas*, which favoured carbon sources are organic acids like acetate, lactate, succinate or citrate (Park *et al*., 2020). Unlike the models where the proteins of PTS participate in modulating genes at the transcription level, the mechanism of action of CCR in *Pseudomonas* is at the mRNA translation level. By directly attaching to A‐rich motifs in the vicinity of the ribosomal binding site (RBS), the Hfq protein (host factor for Q‐beta bacteriophage) represses the translation of catabolite mRNA targets of non‐preferred substrates. The catabolic repression control protein (Crc) interacts with Hfq, generating a stable Hfq‐Crc‐mRNA complex avoiding the translation of the mRNA target. To escape of CCR, small regulatory RNAs (sRNAs) CrcY or CrcZ bind the Hfq‐Crc complex, sequestering both proteins and allowing the translation of mRNA targets (Bharwad and Rajkumar, 2019).

*Pseudomonas* has a vast capacity to produce secondary metabolites. Among them, phenazines, siderophores, 2,4‐diacetylphloroglucinol and lipopeptides can be cited (Yan *et al*., 2017; Shahid *et al*., 2018). Pyocyanin (PYO) is an antibiotic of the phenazines class, which confers a green colour to these strains (Huang *et al*., 2012). PYO biosynthesis starts with the transformation of chorismate to produce phenazine‐1‐carboxylic acid (PCA). Then, by a methyltransferase (PhzM) and a monooxygenase (PhzS), PCA is transformed to PYO. In the presence of succinate, Huang *et al*. (2012) found that Crc binds to two A‐rich sequences in the phzM mRNA, inhibiting PYO biosynthesis. Proteomic studies of *Pseudomonas putida* Hfq null‐mutants grown in succinate revealed that pyoverdine biosynthetic genes are downregulated by this carbon source. However, the specific mechanism by which it occurs is still unknown (Sánchez‐Hevia *et al*., 2018).

### Carbon source regulation of secondary metabolite production in Gram‐positive bacteria

According to their genome guanine‐cytosine (GC) content, Gram‐positive bacteria can be classified in high‐GC bacteria, which holds more than 50% of these nucleotides. Actinobacteria is a representative phylum of this group that comprises the well‐known class Actinobacteria, including orders like Actinomycetales, Streptomycetales or Micromonosporales (Nouioui *et al*., 2018). The other group comprises the low‐GC bacteria, which contents less than 50% of GC and whose representative phylum is Firmicutes that includes the class Bacilli, which covers the order Bacillales or Lactobacillales (Krieg, 2015). The classes mentioned above are essential because they produce most secondary metabolites used in medicine (antibiotics, antifungal, anticancer and immunosuppressive drugs). In their synthesis, participate different pathways like polyketide synthases (PKS), non‐ribosomal (NRPS) and ribosomal peptide synthetases or terpene synthases (TS), among others (Tobias and Bode, 2019).

### Gram‐positive bacteria with low GC content

#### Bacillales

The mechanism of CCR has been studied in some Firmicutes like *Bacillus*, *Staphylococcus*, *Streptococcus* and *Lactobacillus* in which the PTS plays a central role. Contrary to Gram‐negative, *Bacillus* possess an HPr kinase/phosphorylase (HprK/P) that phosphorylates HPr at Ser‐46 when grown in glucose (Castro *et al*., 2009). However, HprK/P dephosphorylates P‐Ser‐HPr when the bacteria are grown in media lacking sugars (Singh *et al*., 2008). These two activities are modulated by some metabolites like fructose 1,6‐bisphosphate, ATP or PP, which trigger the kinase activity, whereas Pi activates the phosphorylation. P‐Ser‐HPr binds to the catabolite control protein A (CcpA), attaching to catabolite‐responsive elements (*cre*), which overlap the promoters of genes sensitive to CCR like *amyE*, *acuA*, *bglP* or *phoP* (Fujita, 2009).

*Bacillus subtilis* has a broad capacity to produce secondary metabolites. Some of them are used as surfactants like the cyclic lipopeptides (iturin, fengycin and the surfactin families). Others like the macrolide (macrolactin) or dihydroisocoumarins (amicoumacins, bacilosarcins, damxungmacin or hetiamacins) and the linear lipopeptides (gageostatins, gageoterins and gageopeptides) have antibiotic activity (Kaspar *et al*., 2019).

Despite the enormous amount and variety of secondary metabolites produced by the *Bacillus* genus, there are very few reports on the effect of the carbon source on their production. In *B. amyloliquefaciens* AR2, the higher production of surfactant occurs in sucrose, followed by glucose. In this case, sorbitol was the worst carbon source for surfactant biosynthesis (Singh *et al*., 2014). In *Bacillus cereus,* glucose negatively influences the enterotoxins HBL (haemolytic) and Nhe (non‐haemolytic) production. However, under anaerobic conditions, its synthesis is activated by fructose (Ouhib‐Jacobs *et al*., 2009).

#### Lactobacillales

The mechanism of CCR has been studied in some members of Firmicutes like *Bacillus*, *Staphylococcus*, *Streptococcus* and *Lactobacillus* in which PTS plays a central role. Contrary to Gram‐negative, *Bacillus* possess an HPr kinase/phosphorylase (HprK/P) that phosphorylates HPr at Ser‐46 when they are grown in glucose. However, HprK/P dephosphorylates P‐Ser‐HPr when the bacteria are grown in media lacking sugars (Singh *et al*., 2008). These two activities are modulated by some metabolites like fructose 1,6‐bisphosphate, ATP or PP, which trigger the kinase activity, whereas Pi activates the phosphorylation. P‐Ser‐HPr binds to the catabolite control protein A (CcpA), attaching to catabolite‐responsive elements (*cre*), which overlap the promoters of genes sensitive to CCR like *amyE*, *acuA*, *bglP* or *phoP* (Fujita, 2009).

The mechanism of CCR has been studied in some members of Firmicutes like *Bacillus*, *Staphylococcus*, *Streptococcus* and *Lactobacillus* in which PTS plays a central role. Contrary to Gram‐negative, *Bacillus* possess an HPr kinase/phosphorylase (HprK/P) that phosphorylates HPr at Ser‐46 when they are grown in glucose. However, HprK/P dephosphorylates P‐Ser‐HPr when the bacteria are grown in media lacking sugars (Singh *et al*., 2008). These two activities are modulated by some metabolites like fructose 1,6‐bisphosphate, ATP or PP, which trigger the kinase activity, whereas Pi activates the phosphorylation. P‐Ser‐HPr binds to the catabolite control protein A (CcpA), attaching to catabolite‐responsive elements (*cre*), which overlap the promoters of genes sensitive to CCR like *amyE*, *acuA*, *bglP* or *phoP* (Fujita, 2009).

### Gram‐positive bacteria with high GC content

#### Actinobacteria

Actinobacteria produce numerous secondary metabolites with broad chemical diversity and biological activity as they can display anticancer, antifungal, antiparasitic and immunosuppressive effects (Traxler and Kolter, 2015; Van der Heul *et al*., 2018). Around 40% of all known commercial natural compounds are produced by actinobacteria, being the *Streptomyces* genus, the most prominent secondary metabolite producer (Bérdy, 2012; Chaudhary *et al*., 2013; Van der Heul *et al*., 2018). The latter is because nearly 5% of the genome in streptomycetes (between 23 and 30 gene clusters) codes to synthesize these metabolites (Ikeda *et al*., 2003).

Due to this extraordinary ability as secondary metabolite producers, the order of Corynebacteriales and Streptomycetales deserves special attention.

### Carbon regulation on secondary metabolites in *Streptomyces*

*Streptomyces* is the most prolific source of secondary metabolites with a wide range of biological activities (Van der Heul *et al*., 2018). The production of secondary metabolites results from a series of global gene expression changes generally preceded by environmental variations or nutrient limitations that lead to morphological differentiation (McCormick and Flärdh, 2012).

These molecules are necessary to complete the *Streptomyces* lifecycle firstly because they must compete with other microorganisms for the nutrients present in the environment. Therefore, they act as defence or signalling molecules in ecological interactions (Osbourn, 2010). Secondly, secondary metabolites also function for self‐toxicity as programmed cell death signals during morphological differentiation (Tenconi *et al*., 2018).

*Streptomyces coelicolor A3(2*) is the genetically best‐known representative of the genus. It has served as a model strain for understanding the actinobacteria chemistry, its biology, antibiotic production and its control. This strain produces three‐coloured antibiotics, and this property facilitates their study at the biochemical and molecular levels. One is actinorhodin (ACT), an aromatic polyketide blue pigment. The other is undecylprodigiosine (RED), a red tripyrolle pigment (Romero‐Rodríguez *et al*., 2016b). The third one is the yellow pigment polyketide known as coelimycin (CPK for cryptic polyketide) (Romero‐Rodríguez *et al*., 2016b; Bednarz et al., 2019). The strain also produces two more antibiotics, the calcium‐dependent (CDA) and the plasmid‐encoded methylenomycin (MM) (Van der Heul *et al*., 2018). All the genes aimed to produce those antibiotics are present in groups constituting the ‘biosynthetic gene clusters’ (BGCs), which typically harbour resistance gene(s) and one or more transcriptional regulators to control biosynthesis. Genome mining analyses in *S. coelicolor* revealed more than 20 BGCs for secondary metabolites (Bentley *et al*., 2002).

The onset of biosynthesis of secondary metabolites is responsive to environmental cues like temperature, light, pH, concentrations of phosphate and oxygen, and the type and levels of the carbon and nitrogen sources (Martín, 2004; Sánchez and Demain, 2002). Hence, secondary metabolite production is tightly regulated by all those environmental and nutritional factors, frequently through intricate signalling cascades that regulate pathway‐specific switches (Liu *et al*., 2013; Romero‐Rodríguez *et al*., 2017). Among these mechanisms, CCR is one of the most conserved. It protects the cells against wasting protein‐synthesizing machinery, which also controls secondary metabolism (Romero‐Rodríguez *et al*., 2016b; Van Der Heul *et al*., 2018). The phrase ‘too much of a good thing can be bad’ is correct when we talk about the *Streptomyces* genus, where glucose is usually the preferred carbon source for growth, but at high concentrations interferes with the formation of secondary metabolites and morphological differentiation (Demain, 1989).

There are reports of more than 30 examples of secondary metabolites suppressed by different carbon sources like maltose, mannose, sucrose and xylose, being the most studied glucose and glycerol (Romero‐Rodríguez *et al*., 2017).

**Fig. 1:**
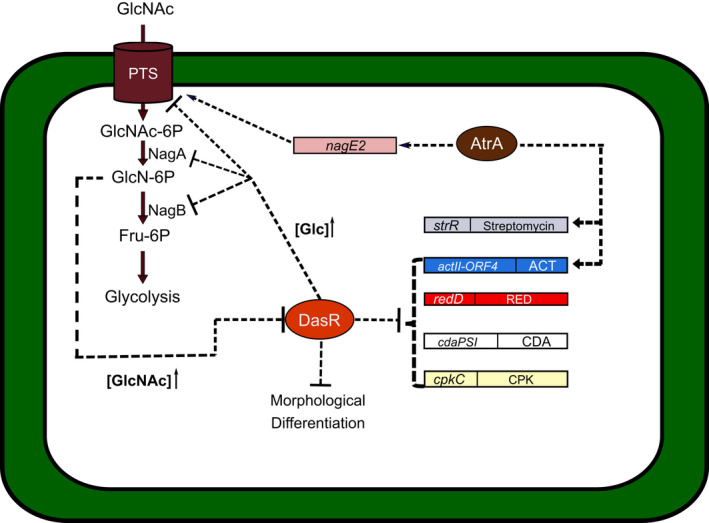
Involvement of the PTS, DasR and AtrA in the *Streptomyces* CCR. GlcNAc transport occurs via PTS which also phosphorylates it. When GlcNAc is at a high concentration, Nag A deacetylates GlcNAc‐6P obtaining GlcN‐6P, which negatively modulates DasR activity. Nag B converts GlcN‐6P into Fru‐6P, which can flow in the glycolysis pathway. At high glucose concentration DasR negatively affects NagA and NagB activities. DasR also represses the genes of morphological differentiation and secondary metabolites production (streptomycin, actinorhodin [ACT], undecylprodigiosin [RED], calcium‐dependent antibiotic [CDA] and coelimycin [CPK]). On the contrary, AtrA acts as an antagonist of DasR since it activates ACT, streptomycin production and GlcNAc transport.

Both, in Gram‐negative and low‐GC Gram‐positive bacteria, certain enzymes from the PTS play pivotal roles in the CCR exerted by glucose (Deutscher *et al*., 2006). In contrast, in *S. coelicolor*, the genes encoding the general phosphotransferases EI (*pstI*), IIACrr (*crr*) and HPr (*pstH*), as well as the permeases NagE1 and NagE2, contain the PTSNag operon which is induced by N‐acetylglucosamine (GlcNAc) (Kamionka *et al*., 2002; Nothaft *et al*., 2003). Under rich‐nutrient conditions, accumulation of GlcNAc promotes growth and blocks developmental processes, while under low nutrient conditions, it favours the development and production of secondary metabolites (Rigali *et al*., 2008). During its transport, GlcNAc is phosphorylated by IIBGlcNAc to GlcNAc‐6P. This signal molecule acts as a negative allosteric effector of DasR (Fig. 1), a GntR family transcriptional factor that controls the GlcNAc transport genes, morphological differentiation and secondary metabolism (Rigali *et al*., 2006; Magdalena *et al*., 2012). Its targets include the antibiotic regulatory gene actII‐ORF4 (van Wezel and McDowall, 2011) and some genes related to the synthesis of coelimycin and CDA (Swiatek‐Polatynska *et al*., 2015). However, the absence of HPr does not affect CCR by glucose, and PTS only responds to the presence of fructose and GlcNAc (Kamionka *et al*., 2002). Likewise, DasR signalling is indirectly under the control of AtrA, a TetR family of transcriptional factors highly conserved in streptomycetes (Nothaft *et al*., 2010). AtrA binds to the promoter of nagE2, activating its transcription and the expressed protein, allows GlcNAc transport. Then GlcNAc phosphorylation produces GlcN‐6P, which, as mentioned before, inhibits DasR. AtrA positively regulates the production of ACT (in *S. coelicolor*) and streptomycin (in *S. griseus*) by controlling *actII‐ORF4* and *strR* respectively (Uguru *et al*., 2005; Hong *et al*., 2007). Thus, AtrA and DasR, at least in *S. coelicolor*, have antagonistic activities (Urem *et al*., 2016).

**Fig. 2:**
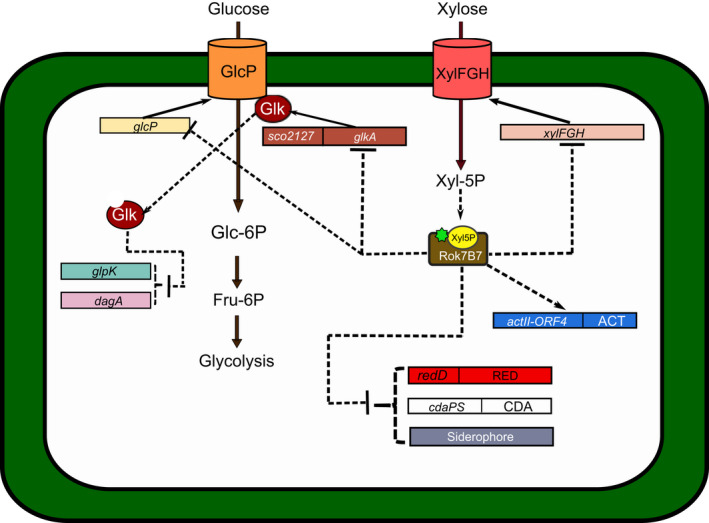
The roles of glucose, Glk and Rok7B7 in the *Streptomyces* CCR. Glucose transport occurs by the symporter GlcP, which has been proposed to interact with Glk, eliciting its modification. The modified Glk exert CCR on the uptake and metabolism of other carbon sources like glycerol (*glpK*) or agarose (*dagA*). Xylose is transported and phosphorylated by XylFGH to produce Xyl‐5P. This compound activates the regulatory protein Rok7B7 to repress the expression of *glcP*, *xylFGH* (xylose uptake*) glkA* and the production of the secondary metabolites RED, CDA and siderophores. On the contrary, it stimulates ACT production.

The mechanism of the glucose CCR in *Streptomyces* remains unknown (Titgemeyer *et al*., 1995). Likely, CCR responds to carbohydrate metabolism intermediaries (Ramos *et al*., 2004; Borodina *et al*., 2008) and the enzyme glucose kinase (Glk) (Angell *et al*., 1992, Kwakman and Postma, 1994; Romero‐Rodríguez et al., 2015a) (Fig. 2). Glk initiates the glucose catabolism by catalysing its phosphorylation to glucose 6‐phosphate (Kawai *et al*., 2005). Angell *et al*. (1992) reported a correlation between Glk and CCR in *S. coelicolor* mutants lacking the *glkA* gene. These mutants can then use glycerol, arabinose, fructose and galactose in the presence of glucose. Their complementation with the *glk* gene from *Zymomonas mobilis* led to Glk activity recovery, the ability to grow in glucose but not to glucose repression (Angell *et al*., 1994). When *glkA* mutants are grown in galactose and glycerol, the glycerol kinase and agarase activities are relieved of CCR. These results reinforced the theory that Glk plays an essential and pleiotropic role in CCR, besides participating in the metabolic flow (Kwakman and Postma, 1994).

Gubbens *et al*. (2012) carried out a proteomic correlation between the wild type *S. coelicolor* M145 strain and a *glkA* null‐mutant to understand the effect of Glk on CCR. Thus, these authors identified Glk‐dependent and independent carbon control pathways, suggesting that Glk is not the only responsible factor for the CCR. In this and other analyses, Rok7B7 (a member of the ROK family regulators) and XylFGH (the xylose uptake operon) were upregulated by glucose through an independent way of Glk (Romero‐Rodríguez *et al*., 2016a). In *S*. *coelicolor* Rok7B7 negatively regulates the adjacent operon *xylFGH* (Swiatek *et al*., 2013; Lu *et al*., 2020), promotes ACT production and suppresses both RED and CDA biosynthesis (Fig. 2), probably by direct binding to *actII‐ORF4* and *redZ* promoters (Park *et al*., 2009). Interestingly, DasR and Rok7B7 repress the expression of Glk (Swiatek‐Polatynska *et al*., 2015), while Glk represses Rok7B7 suggesting it might play a compensatory role in CCR in the presence of xylose but in the absence of glucose (Gubbens *et al*., 2012). Together with the fact that the absence of *rok7B7* causes CCR loss, it directly involves Rok7B7 in the CCR effect (Gubbens *et al*., 2012; Romero‐Rodríguez *et al*., 2016a). A recent report shows that a Rok7B7 orthologue of *S. avermitilis* (*sav_2248*) binds to the promoter regions of *ave* structural genes, leading to repression of avermectin production by an independent pathway of *aveR* (a LuxR‐family activator). Besides, it promotes oligomycin A production by binding to the activator gene *olmR1* (another LuxR‐family activator). In the presence of glucose, the affinity of Rok7B7 for its target site on the promoter of *xylFGH* increases repressing xylose uptake. This carbon source, in turn, acts as an inducer releasing the promoter from binding by Rok7B7. Additionally, eleven new target genes of Rok7B7 involved in the development, secondary metabolism, glucose uptake and primary metabolic processes have been identified in this microorganism (Lu *et al*., 2020) (Fig. 2).

To separate the GlkA and glucose effects, Romero *et al*. (2016a) studied the wild type *S*. *coelicolor* M145 strain and a *glk* null‐mutant (Δ*glkA*) complemented with the *glk* gene from *Z. mobilis*. The wild type strain was grown in glucose (repressive conditions), and its transcriptome compared to this same strain grown in agarose as the only carbon source (non‐repressive conditions). These authors also compared the wild type strain transcriptome to that of the Δ*glkA* strain grown in glucose. Here, the presence of glucose was able to regulate 32 transcriptional regulators and four two‐component systems. Hence, in this study, it was possible to determine that although Glk plays a role in CCR, glucose also significantly affects this mechanism (Romero‐Rodríguez *et al*., 2016a).

**Fig. 3:**
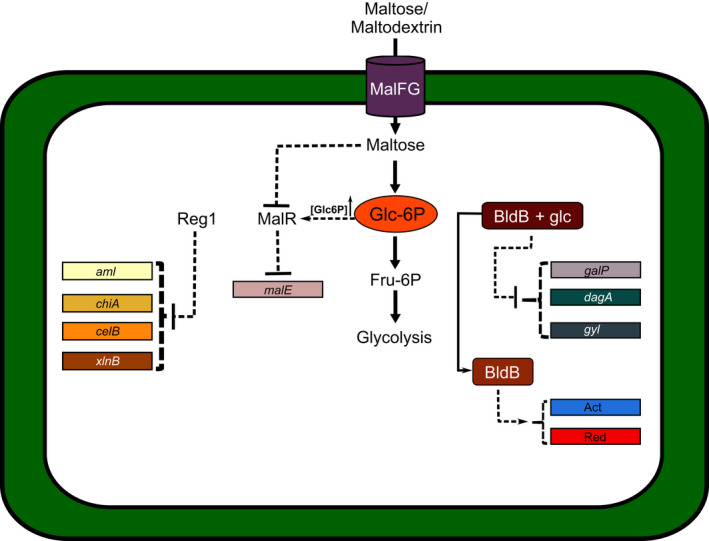
Roles of the MalR, Reg1 and BldD regulators in the *Streptomyces* CCR. MalFG transports maltose and maltodextrin. Internal maltose concentrations inhibit the specific regulator MalR which represses the gene *malE* (maltose‐binding protein) for maltose utilization. Maltose is hydrolysed to glucose and converted to Glc‐6P. High concentrations of this metabolite activate MalR, which subsequently exerts CCR on *malE*. The orthologous protein of MalR in *S. lividans* is Reg1. This protein represses the expression of genes encoding for hydrolytic enzymes like amylase (*aml*), chitinase (*chiA*), cellulase (celB) and xylanase (*xlnB*). The morphological differentiation protein BldB is also involved in the production of ACT and RED. When glucose is present, BldB suppresses the use of other carbon sources such as galactose (galP), agarose (dagA) and glycerol (gyl).

Engineering transcriptional regulators represent a useful approach to modify carbon flux for the overproduction of metabolite intermediates (Romero Rodríguez et al., 2015b). Since most of them remain uncharacterized, their study may give groundwork for improving production levels or unveiling secondary metabolites (Romero‐Rodríguez *et al*., 2016a). The protein Reg1 (a LacI/GalR family transcriptional factor) is essential in the regulatory mechanisms of *S*. *lividans*. Its disruption has a pleiotropic effect by releasing genes encoding amylase and chitinase from CCR (Nguyen *et al*., 1997). In *S*. *coelicolor*, the MalR repressor (Fig. 3), which is 95% identical to Reg1, regulates the maltose utilization, gene *malE*. Reg1 also binds to CCR sensitive genes like chitinase, xylanase and cellulase (Nguyen, 1999). In the ACT biosynthetic pathway of *S. coelicolor*, glucose represses a protein involved in the acetyl‐CoA‐malonyl‐CoA condensation, and the *sco5071* gene, which is a putative regulator of tailoring enzymes of the same pathway (Romero‐Rodríguez *et al*., 2016b). This sort of information opens new opportunities for rational engineering of essential proteins within biosynthetic pathways.

Proteins involved in morphological differentiation are also affected by CCR, like *bld* (bald) genes, implicated in aerial hyphae formation (Eccleston *et al*., 2002). Mutants lacking *the bldB* gene no longer show glucose repression. On the contrary, its overexpression blocks sporulation initiation (van Wezel and McDowall, 2011). Other *bld* mutants showed decreased production of antibiotics and defective development of aerial mycelium when grown in glucose. Mutants in *bldA, bldG, bldH* and *bldK* sporulate well when grown in mannitol or glycerol and other *bld* mutants (*bldC, bldH*) showed good sporulation. This finding indicates that for most known *bld* mutants, there is a way to overcome their mutations and continue their healthy development (van Wezel and McDowall, 2011).

Despite the great efforts made to explain the complex regulatory network of CCR in *Streptomyces*, it has not yet been possible to have a model that thoroughly explains it. Further research is needed to unite the pieces of the puzzle to understand this phenomenon. Recently we detected a direct connection between a two‐component system formed by a kinase and a response regulator, with Act and Red production in *S. coelicolor*. Therefore, it may be the link between sugar catabolism and secondary metabolite production (R. Cruz‐Bautista, A. Zelarayan, B. Ruiz‐Villafán, R. Rodríguez‐Sanoja and S. Sánchez, in preparation).

### CCR in other actinobacteria

The genus *Saccharopolyspora* produces various chemical structures like macrolides, quinones, alkaloids, peptides and glycolipids (Sayed *et al*., 2019). Erythromycin A is a macrolide discovered in 1949 currently produced at a large‐scale by the bacteria *Saccharopolyspora erythraea* (Wright *et al*., 2014). Surprisingly, there are no cluster‐situated regulatory (CSR) genes near the erythromycin biosynthesis cluster (Liao *et al*., 2015). However, reports show how the transcription levels of genes for the biosynthesis of the precursors propionyl‐CoA and methylmalonyl‐CoA influence erythromycin production (Li *et al*., 2013).

**Fig. 4:**
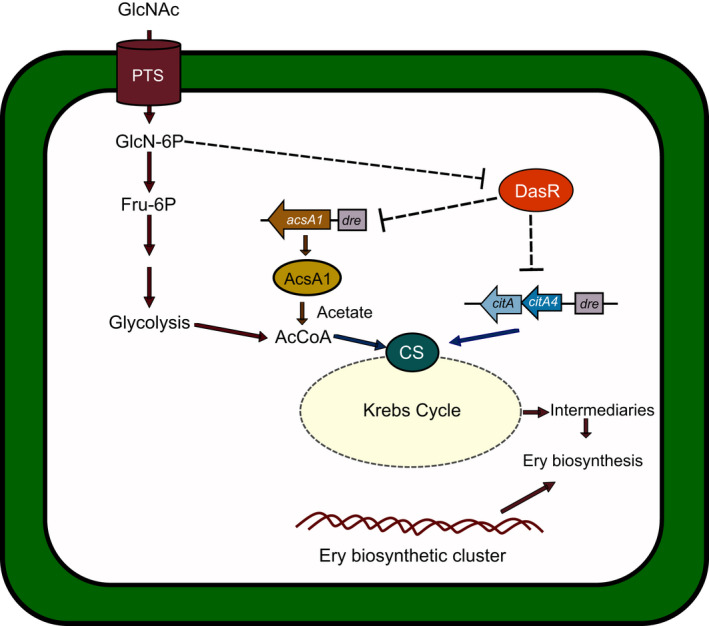
The negative effect of the carbon source in *Saccharopolyspora erythraea*. GlcNAc is transported by PTS converting it to GlcNAc‐6P. Deacetylation of this compound produces GlcN‐6P, an inhibitor of DasR activity. On the contrary, when other carbon sources different from GlcNAc are present, DasR binds to its *dre* boxes in genes such as acetate (*acsA1*) and citrate (*citA‐A4*) synthases which affect acetate and citrate assimilation.

*Saccharopolyspora erythraea* has all the necessary genes for chitin and N‐acetylglucosamine utilization (Liao *et al*., 2014). The expression of all those genes is subject to repression by DasR (Fig. 2), which regulates erythromycin synthesis and morphological differentiation (Liao *et al*., 2015). Recently You *et al*. (2018) demonstrated that in the presence of GlcNAc, DasR repressed the transcription of the gene *acsA1* to utilize acetate as the sole carbon source (Fig. 4). DasR, also influences the synthesis of acetyl‐CoA in the cell. Besides repressing the expression of citrate synthase, the Krebs cycle and metabolic rate are indirectly controlled (Liao et al., 2014).

*Saccharopolyspora spinosa* produces spinosins, macrolides used to control plagues like diamondback moth (*Plutella xylostella*), or the beet armyworm (*Spodoptera exigua*) among many other lepidoptera (Wang *et al*., 2014). Spinosad is a mixture formed by a spinosins A and D (Tao *et al*., 2019). Jin *et al*. (2006) previously reported a negative effect of glucose (from 79.6‐115.3 g l^‐1^) on the spinosyns production under submerged fermentation, with 58.8 g l^‐1^ as the optimal glucose concentration. There are reports that, in addition to the use of fed‐batch cultures, under the continuous control of glucose, mannitol can increase the production of spinosyns (Tao *et al*., 2019).

The genus *Corynebacterium* comprises the pathogenic strain *Corynebacterium diphteriae* and some other non‐pathogenic and industrially relevant strains like *Corynebacterium xerosis*. This strain produces secondary metabolites like coryxin, a cyclic polypeptide active against both Gram‐negative (*E. coli* and *Pseudomonas aeruginosa*) and Gram‐positive (*Staphylococcus aureus* and *Streptococcus mutans*) bacteria (Dalili *et al*., 2015). El‐Banna *et al*. (2006) previously reported that the production of antibiotics by *C. xerosis* and *Corynebacterium* was negatively affected by lactose and ribose. In its CCR mechanism, *C. glutamicum* involves the PTS system. This system has genes encoding enzyme I, HPr and four enzymes II permeases, specific for glucose, fructose, sucrose and one unknown substrate (Moon *et al*., 2020).

### Carbon source regulation of secondary metabolite production in fungi

In *Aspergillus flavus*, glucose is the most severe carbon source in CCR as it represses a large portion of enzymes like those for the synthesis of aflatoxin (AF). This mechanism includes a set of genes, *creA*, *creB*, *creC* and *creD* (Boase and Kelly, 2004), acting together and regulating each other, being creA the major transcriptional repressor (Chulkin *et al*., 2011). In *Aspergillus nidulans*, *creA* recognizes and binds to the consensus sequence 5'‐SYGGRG‐3’ on the related gene's promoter region (Kulmberg *et al*., 1993). *creB* appeared to encode a deubiquitinating enzyme (Lockington and Kelly, 2001), and *creC* encodes a protein with a WD‐40 motif, which is bound to *creB* to form a protein complex (Adnan *et al*., 2018). *creA* in *A. nidulans* is present within the nucleus, and its localization does not depend on the absence of either *creB* or *creD* (Lockington and Kelly, 2001), yet the degree of *creB* in the cell is under the control of creD (Roy *et al*., 2008). *creB* and *creC* are in the cytoplasm, and *creD* (proposed to be implicated in ubiquitination), is present in the whole cell (Boase and Kelly, 2004). Gene deletion and overexpression experiments were useful to explain the role of *creA* in morphology, pathogenicity and secondary metabolite production in *A. flavus*, using glucose, sucrose and maltose (Fasoyin *et al*., 2018). A Δ*creA* mutant has a critical development deficiency when grown on complete and minimal media containing maltose. Δ*creA* mutants showed a lower AF production when grown in maltose as the sole carbon source, exhibiting a decrease in the expression of the biosynthetic genes *aflR*, *aflM* at 48 h and *aflD*, *aflO, aflP* at 72 h of growth (Fasoyin *et al*., 2018).

*Penicillium chrysogenum* is the fungus that produces penicillin. This production is negatively affected by glucose, followed by fructose, galactose and sucrose. Glucose represses expression of the biosynthetic *pcbAB* genes (ACV [L‐delta‐(alfa‐n aminoadipyl)‐L‐cysteinyl‐D‐valine] synthetase), *pcbC* (isopenicillin synthetase) and *penDE* (isopenicillin acyltransferase). On the contrary, oligomannuronate and alginate increase their transcription (Ozcengiz and Demain, 2013). In the intergenic region between *pcbAB* and *pcbC* genes in *P. chrysogenum*, six CreA binding sites allowed negative glucose control of penicillin biosynthesis (Cepeda‐García *et al*., 2014). The CreA‐1 binding site has an essential role since its deletion causes a 50% loss of repression under non‐controlled pH conditions. However, when maintained at a value of 6, there is a total loss of the *pcbAB* repression. It is worth mentioning that the *penDE* promoter contains ten CreA binding sites; thus, its regulation by CreA is entirely feasible (Cepeda‐García *et al*., 2014).

Cephalosporin C (CPC) is one of the main beta‐lactams produced in the world. In its production participates the fungus *Acremonium chrysogenum*. Carbon sources allowing rapid growth harm CPC production (Ozcengiz and Demain, 2013). When *A. chrysogenum* is cultured in glucose, the transcription levels of the genes *cefEF* (encoding for an expandase) and *pcbC* (encoding for a cyclase) decreased. This effect is probably due to the *cre‐1* binding sites located upstream of those genes.

Interestingly, glycerol as a carbon source can increase the transcription levels of both *cefT* (beta‐lactams transporter) and *pcbC* genes, reflecting in an increase in CPC production (Shin *et al*., 2010). Studies with glycerol as the carbon source in *A. chrysogenum,* allowed the generation of alternative strategies to produce CPC. Thus, the use of extracts from the algae *Chlorella pyranoidosa*, which contains carbohydrates, proteins and lipids, is a good example. The culture medium with algae extracts and supplemented with 6% glycerol increased antibiotic production (Lee *et al*., 2017).

In several species of fungi, the pyruvate dehydrogenase complex (PDH) is relevant for morphology, pathogenicity and carbon source utilization (Kolobova *et al*., 2001). The complex is under the pyruvate dehydrogenase kinases (PDHK) and phosphatases (PDHP) direction.

The central role of the three PDHKs, PkpA, PkpB and PkpC occurs at the carbon source consumption. PkpA and PkpB are mitochondrial, while PkpC location in the mitochondria is a carbon source‐dependent process (Patel and Korotchkina, 2006). Only PkpA appeared to control PDH activity. *pkpA* and *pkpC* deleted mutants grown in glucose show a decreased glucose utilization and a relief of CCR. Likely, this phenotype is due to glycolysis and tricarboxylic acid cycle (TCA) pathways deregulation. Moreover, PkpC has a significant role in using preferred (glucose) and alternative (cellulose, acetic acid) carbon sources by regulating carbon source‐specific metabolic progression and enzyme secretion (Ries *et al*., 2018).

Citrinin is a toxic secondary metabolite isolated from *Penicillium citrinum*. Besides its antibiotic, antifungal and antiprotozoal activities, it also shows hepato‐ and nephrotoxicity (Li *et al*., 2017; Xu *et al*., 2018). Unlike other microorganisms, *P. citrinum* produces high citrinin levels in the presence of glucose, compared to sucrose. According to transcriptomic studies, when *P. citrinum* grows in glucose, it reorients its primary metabolic pathways. Therefore, it drives the carbon to increase the acetyl‐CoA and malonyl‐CoA levels, resulting in a higher concentration of precursors for polyketide synthesis. Similarly, the PKS genes involved in secondary metabolism and citrinin biosynthesis undergo upregulation in the glucose‐cultured *P. citrinum* (Li *et al*., 2017).

Lovastatin and its derivatives (statins) are of worldwide interest in treating hypercholesterolaemia. These compounds inhibit the enzyme hydroxymethylglutaryl coenzyme A (HMG‐CoA) reductase (Mulder *et al*., 2015). Sequencing studies show that its biosynthetic cluster comprises 18 open reading frames (ORFs) from which *lovE* and *lovH* are regulatory proteins. Interestingly, both genes have putative sequences for binding the regulatory CreA protein (Hajjaj *et al*., 2001).

## Concluding remarks

Throughout history, humanity has experienced numerous deaths due to different microbial infections. Hence the importance of the isolation of new compounds with antimicrobial activity. Microorganisms are remarkable producers of natural products and amazing producers of secondary metabolites (Sánchez and Demain, 2017), comprising half of the pharmaceuticals on the market today. Among them, antibiotics have played an essential part in treating infectious diseases and saved many human lives. Of the 38,000 biologically active compounds reported to be produced by microbes, 42% are from actinobacteria, 47% from fungi and 12% from unicellular bacteria (Berdy, 2015). However, most of the secondary metabolites currently on the market come primarily from the genus *Streptomyces*. This fact is likely due to the harsh habitat from which they live and the strategies they must have to survive, in addition to their particular life cycle. In these hostile environments, *Streptomyces* usually face a lack of some nutrients, which generally triggers secondary metabolite production, in coordination with morphological differentiation. Among these nutrients, the source of carbon, nitrogen and phosphorus play an essential role. This review aimed to deep into recent advances of regulation by the carbon source. Although described in *E. coli* almost 60 years ago, the *Streptomyces* carbon source regulation precise mechanism remains unveiled. These regulatory mechanisms are of general knowledge in other microbial groups, including Gram‐positive bacteria with low G‐C content, Gram‐negatives like *Pseudomonas*, and fungi. For *Streptomyces,* the Glk, Rok7B7, BldB and other specific regulators like MalR or Reg1 participate in this process (Ruiz‐Villafán et al., 2014). However, a complete panorama of CCR in *Streptomyces* is still absent. Especially relevant for this genus is the Glk role in the CCR mechanism and its influence on different targets (Romero‐Rodríguez *et al*., 2015a). However, in the absence of DNA binding motifs, which prevent binding to promoter regions of those genes (Angell *et al*., 1992), Glk should utilize alternative ways to exert its action. One of them likely occurs by influencing the expression or modification of transcriptional factors. Transcriptome studies showed that Glk influenced nine transcriptional regulators (Romero‐Rodríguez *et al*., 2016b).

The question is, how can Glk split its catalytic from its regulatory functions? Recently, the report of Glk crotonylation by an acyltransferase supported a plausible mechanism to explain how the modified enzyme may interact with its corresponding transcriptional factors (Sun *et al*., 2020).

Although Glk plays a role in CCR, glucose seems to exert the leading role in this mechanism (Romero‐Rodríguez *et al*., 2016a). Transcriptome studies showed that the expression of 40 DNA binding proteins, including 31 transcriptional regulators and four two‐component systems, are regulated by glucose, and probably are involved in the signals and the effects elicited by this carbon source (Romero Rodríguez et al., 2016a). Transcriptional factors stimulated by glucose included the MerR, LacI, TetR, GntR, MarR, AsnC families and sigma factors. On the other hand, transcriptional factors repressed by glucose entered members of the GntR, MarR and DeoR families and sigma and anti‐sigma factors (Romero‐Rodríguez *et al*. 2016a).

Currently, it is imperative to elucidate the link between glucose metabolism and the mechanism of CCR in *Streptomyces* (Titgemeyer *et al*., 1995). Recently, we have found the participation of a two‐component system in the CCR of *S. coelicolor*. Both the histidine kinase activity and the response regulator were strongly affected by glucose repression. CCR responds to carbohydrate metabolism intermediaries like fructose‐6‐bisphosphate and phosphoenolpyruvate, which may serve as ligands between glucose metabolism and CCR (Ramos *et al*., 2004). Among the targets of that response regulator, actinorhodin and the CDA production genes were highlighted (R. Cruz‐Bautista, A. Zelarayan, B. Ruiz‐Villafán, R. Rodríguez‐Sanoja and S. Sánchez, in preparation).

Regarding filamentous fungi, the Cre proteins (mainly CreA) are of great importance for the onset of CCR.

The study and elucidation of the molecular mechanisms of CCR and the advancement of basic science can be relevant for secondary metabolite's industrial production. Therefore, modifications in one or several of these mechanisms are necessary to overcome CCR regulation and obtain mutants with an improved secondary metabolite production capacity.

## Conflict of interest

No potential conflict of interest was reported by the authors.

## Funding Information

The present work was funded by the CONACYT grant A1‐S‐9143, and the NUATEI programme from Instituto de Investigaciones Biomédicas, UNAM. Part of this work was supported by the PAPIIT, DGAPA, grant number IN205519.
